# The Care of the Soldiers in Provincial Hospitals

**Published:** 1914-10-17

**Authors:** 


### The Care of the Soldiers in Provincial Hospitals.

### CAMBRIDGE: THE NEW WAR HOSPITAL.

On Saturday and Sunday last the cloisters at Trinity College, Cambridge, and the tents erected in the grounds at the rear of the college which have been so admirably fitted up and have served so well as the 1st Eastern General Hospital, Cambridge, were dismantled, and the wounded were in a most marvellous and rapid manner removed to that portion of the new hospital which has been erected on the Clare College cricket ground in Burrell's Walk, Cambridge. The patients were none the worse for this removal.

A large number of the wounded sailors and soldiers continue to attend the special electrical and $x$-ray department at Addenbrooke's Hospital to be skiagraphed. The assistance which Addenbrooke's Hospital has been able to render in this direction has been a great advantage to the 1st Eastern General Hospital and to the physicians and surgeons who are responsible for the care of the wounded.

It will be remembered that, as recorded in previous issues, the new 1st Eastern General Hospital which, as stated, is on Clare College cricket ground, a part of which has been completed in remarkably short time, and is already occupied by the wounded transferred from Trinity College, was originally intended to accommodate 500 patients; but it is now understood that the hospital, when quite completed, will have sufficient accommodation for the reception of at least 1,000 wounded sailors and soldiers.

It is hoped that it will be possible in the near future, or at least as soon as completed, to give a detailed account of the hospital and the accommodation which it affords, and to reproduce for the benefit of our readers the block plans of same.

### CARDIFF: WHERE THE MEN LIKE TO CONVALESCE.

The work of treating the wounded soldiers admitted from the Expeditionary Forces and cases belonging to home troops continues successfully at the 3rd Western General Hospital, Cardiff. Of the first convoy of men from the Front eighty-five have been discharged, and forty of the second, making 125 discharges out of 240 admissions. Some of the wounds have been of a severe nature, but all the patients are progressing to the extent expected. The percentage of $x$-ray cases here is about sixty. The only death recorded was that of a member of a Territorial unit from pneumonia.

The public have shown great interest in the men's welfare, gifts been many, useful, and varied; entertainments are provided weekly, and services are held by the different denominations on Sundays. The men's cheeriness and gratitude are undoubted. Not a few country houses are at the disposal of the authorities for the receipt of convalescents, but it is almost invariably found that the soldier prefers to spend his sick furlough at home.

Three of the members of the resident medical staff of King Edward VII.'s Hospital have been called up, and six nurses of the 3rd Western General Hospital have left for service abroad.

### COVENTRY: THE FIRST BATCH OF WOUNDED.

The Coventry and Warwickshire Hospital received the first batch of twenty wounded soldiers from the 1st Southern General Hospital, R.A.M.C.T., Birmingham, on Wednesday, last week.

The men were conveyed by motor-car, and arrived in time for tea. The patients are suffering principally from the effects of gunshot wounds in the arms, head, and legs, and after a short period of treatment will be fit to be discharged to a convalescent home or to their regimental depôts.

### GRAVESEND: BELGIAN WOUNDED ARRIVE.

No further convoy of wounded has arrived at the Gravesend Hospital. Some of the first to arrive are now being discharged as convalescent, and are taking away with them as souvenirs shrapnel bullets and pieces of shell which have been extracted. The hospital has been asked to receive Belgian wounded, and the first batch is expected at any moment.

The Gravesend and Northfleet Voluntary Aid Detachment is mobilised, but so far no patients have been received into their temporary hospital of fifty beds. They have, however, been called upon to prepare a small hospital at All Hallows (on the Isle of Grain) for the sick soldiers from the Thames and Medway forts of Grain, Slough, and Cliffe. This work they carried out at short notice, and very quickly were tending patients there.

### LEICESTER: THE COUNTY REGIMENT.

The fourth batch of wounded soldiers from the Front was received at the 5th Northern General Hospital, Leicester, on Wednesday, last week. Numbering 125 sick and wounded, the party was despatched from Southampton by ambulance train in charge of Captain Stracey, and met with a hearty reception on arrival. Under the supervision of Mr. A. W. Faire and Mr. W. F. E. Seymour the arrangements for the transit of the patients was expeditiously arranged without hitch, Mr. Reggie Corah again acting as marshal to the fleet of motor-cars kindly lent by the members of the Leicestershire Automobile Club. The wounded men, among whom were some from the Leicestershire Regiment, were incapacitated in the course of the long battle of the Aisne, but all are reported as progressing satisfactorily. During the week two batches of patients have been transferred to the Nottingham General Hospital, while others have been discharged to their depôts or on furlough. Additional nurses have been called up for the service of the institution.

The Mayoress of Leicester's Equipment Fund for the provision of useful articles for the soldiers now amounts to £670. 2,300 useful articles of clothing have been supplied to the Base Hospital, 135 for No. 12 ambulance train, 80 rugs for the transport of wounded soldiers, and £50 has been donated to the County Director of Voluntary Aid Detachment Fund towards the purchase of a new motor ambulance.

Messrs. W. P. Hogg and George Wilson, the first and second house surgeons to the Leicester Royal Infirmary, have been mobilised for duty with the R.A.M.C., for which they volunteered, and are in residence at Seaford Camp.

### SHEFFIELD: THE AMUSEMENTS OF CONVALESCENCE.

The cases sent here for attention in the 3rd Northern General Hospital are making remarkably rapid progress towards convalescence. Although within five minutes' motor-ride from the railway stations, the Training College, a modern structure, well lighted and airy, standing in its own spacious grounds, is situated in one of the city's pleasantest West-end suburbs, where the smoke from the East-end works is rarely felt. In the fine autumn weather which has been enjoyed during the past week most of the patients have made great progress, and they have been able to spend long days in the open air.

Already three convoys of over 120 each have been dealt with. Each convoy has comprised cases of great gravity, but, happily, only one death has yet been reported—that of Private Dowling, of the 1st Lincolnshire Regiment, whose home was at West Ham. He was very severely wounded, both in the arms and legs, and amputation had to be resorted to. In fact, his case was regarded as hopeless from the first. Two other very serious cases were included in the third convoy, which reached Sheffield on the 3rd inst., but these have during the last few days been progressing satisfactorily. The last convoy included about forty "bed" cases, but many of these have since been able to get about and take part in some of the lighter games and recreations, of which there is lavish provision. For the more robust football easily holds first place, while for those not yet able to take such vigorous exercise quoits are a popular diversion. Those who are still confined to the wards find a game of cards or dominoes very acceptable, but at present a craze for "jig-saw" puzzles has spread through the hospital.

Thanks to the organising capabilities of Mr. F. J. O. Coddington, a barrister practising in Sheffield, an array of voluntary musical talent, sufficient for two or three concerts per week throughout the winter, has been assured. Hardly less remarkable than the quantity is the quality of the response to Mr. Coddington's appeal. Prominent professional musicians, eminent local singers and instrumentalists, quartet parties, small orchestras, soloists on all types of instruments, choirs, glee-singers, hand-bell ringers, operatic combinations, reciters, a juvenile prodigy—all offer to help with generous whole-heartedness. In addition to this local response, the soldiers are, thanks to the generous co-operation of the management and artistes of the Sheffield Empire and Hippodrome, assured of a weekly afternoon performance by variety artistes. On Saturday nights there is to be a "sing-song," at which the programmes will be of a less formally musical character.

Needless to say, these efforts have met with the warmest possible appreciation on the part of the wounded men, who have sought opportunity to give expression of their thanks, not only to the organisers and artistes, but to the officers in charge of the hospital. These entertainments coupled with the innumerable gifts of every imaginable form of delicacy and comfort and means of recreation for the men, have given them an impression of what a Yorkshire welcome really means, and, as may be imagined, convalescence comes, in one sense, all too soon for many of the wounded.

### TORBAY HOSPITAL AND WOUNDED BELGIANS.

Last Sunday the Mayor of Torquay received a wire from London inquiring if a Torquay hospital could receive fifty Belgian wounded soldiers on Wednesday, the 14th inst. These soldiers were coming with others from Ostend, and were brought to England by the Admiralty. This hospital has decided to receive the fifty soldiers, and special wards are now being prepared for their reception.

### TUNBRIDGE WELLS: NEW ARRIVALS AND NEW FUNDS.

Ten men of the first twenty-four wounded received into the General Hospital were discharged cured on Saturday, October 10, and conveyed to Fort Pitt, Chatham, in private motor-cars, there to be fitted out with their usual uniforms and then to go to their homes for a furlough before returning to the Colours again. The cars returned to Tunbridge Wells with fifteen wounded men who had only returned from the Front the day before. It was known that a larger number would be returning in the cars than were sent to Chatham, therefore a sufficient number of cars were sent. Of the cases, thirteen were for the General Hospital and two eye cases were for the Eye and Ear Hospital at Tunbridge Wells.

The tetanus case in the General Hospital is doing very well indeed, and a complete recovery is expected. The anxiety of this case was largely augmented by the inability of obtaining a good supply of serum, all usual sources of supply being exhausted by the demands of the military authorities. There are a good number of Belgian refugees in Tunbridge Wells and surrounding districts, and more are expected.

Last Saturday night soon after ten o'clock an urgent message was received at the General Hospital from the Horse Guards asking if wounded Belgians could be taken in, as beds were urgently wanted. The reply was that fifteen could be taken in at once. These beds were not in the hospital, but had been promised by a local tradesman, Mr. E. Oliver. He was communicated with and voluntary help obtained, and the beds were carted into the hospital with mattresses, etc., and the emergency beds all ready for the patients by 1.30 A.M. This was managed without disturbing the day staff of the hospital, who were having their well earned repose. Nothing more was heard from the Horse Guards until Sunday night, when a wire came stating that the wounded Belgians were coming in in batches, and if the fifteen beds were wanted due notice would be given.

Two small funds have been started in connection with the wounded at Tunbridge Wells. One with the object of paying for the shaving and hair-cutting of the men whilst in the hospital. The other to be used to defray the travelling expenses of the wives or mothers of the men, most of whom are in quite poor circumstances, and in most cases have to travel some distance to see their husbands or sons.

### WAKEFIELD: THE REFUGEE QUESTION.

Wakefield has now been informed that they should be ready to receive wounded soldiers as soon as any more wounded arrive from a West Yorkshire Regiment, as the War Office prefers to send the wounded as near their own homes as possible. As many as thirty Voluntary Aid Detachment nurses have now received a really thorough training at the hospital here, and many of them have become quite useful helpers.

The great question at present so far as Wakefield is concerned is the reception of Belgian refugees, and arrangements are practically complete for the reception of fifty, fourteen of whom can be accommodated at a convalescent home, which will be most suitable for housing refugees.

One of the hospital honorary medical staff has volunteered to act as medical officer to the refugees, and has been accepted by the committee. Another has received an appointment to the French Red Cross Society.

### WESTON: THE SPECIAL CARE OF CONVALESCENT SOLDIERS.

The Royal West of England Sanatorium, Weston-super-Mare, a convalescent home where hot and cold sea-water baths are made a special feature, has set aside 150 beds free of all charge for the use of wounded or invalided sailors and soldiers, and the institution is registered in the proper quarter. The offer differs from the usual one of taking strictly convalescents in that the authorities are hoping to have sent to them cases requiring special care. The medical and nursing staffs have been increased, so that the greater individual attention required by more acute cases may not be lacking. Arrangements have been made for any number of cases requiring surgical dressings; and ward kitchens, an unheard-of thing in the average convalescent home, have been arranged to serve different sections of wards. The elaborate sea-water bathing plant should be of especial value in many cases, and these will be given daily, if necessary, instead of twice weekly as is usual.

The provision of the above beds has necessitated the non-admission of female patients during the war, but male civilian cases are being received as usual for the present. The loss of income entailed and the additional expense to be incurred through having all, instead of only a small part, of the beds full throughout the winter is being provided for by the hon. lady superintendent, Miss Mawe, who has helped to raise a special fund, which already stands at nearly £1,500. This amount has been chiefly provided by the subscribers to the institution, but there have been also several generous local donations. The local branches of the various relief societies and private sewing parties have generously supplied dressings and clothing.

